# Rising Prevalence and Neighborhood, Social, and Behavioral Determinants of Sleep Problems in US Children and Adolescents, 2003–2012

**DOI:** 10.1155/2013/394320

**Published:** 2013-05-30

**Authors:** Gopal K. Singh, Mary Kay Kenney

**Affiliations:** US Department of Health and Human Services, Health Resources and Services Administration, Maternal and Child Health Bureau, 5600 Fishers Lane, Room 18-41, Rockville, MD 20857, USA

## Abstract

We examined trends and neighborhood and sociobehavioral determinants of sleep problems in US children aged 6–17 between 2003 and 2012. The 2003, 2007, and 2011-2012 rounds of the National Survey of Children's Health were used to estimate trends and differentials in sleep problems using logistic regression. Prevalence of sleep problems increased significantly over time. The proportion of children with <7 days/week of adequate sleep increased from 31.2% in 2003 to 41.9% in 2011-2012, whereas the prevalence of adequate sleep <5 days/week rose from 12.6% in 2003 to 13.6% in 2011-2012. Prevalence of sleep problems varied in relation to neighborhood socioeconomic and built-environmental characteristics (e.g., safety concerns, poor housing, garbage/litter, vandalism, sidewalks, and parks/playgrounds). Approximately 10% of children in neighborhoods with the most-favorable social environment had serious sleep problems, compared with 16.2% of children in neighborhoods with the least-favorable social environment. Children in neighborhoods with the fewest health-promoting amenities or the greatest social disadvantage had 37%–43% higher adjusted odds of serious sleep problems than children in the most-favorable neighborhoods. Higher levels of screen time, physical inactivity, and secondhand smoke exposure were associated with 20%–47% higher adjusted odds of sleep problems. Neighborhood conditions and behavioral factors are important determinants of sleep problems in children.

## 1. Introduction

Sleep problems in children have significant impacts on their health and well-being [1–4]. Inadequate sleep in children has been shown to be associated with poor academic performance, behavioral problems, poor mental and physical health, obesity and weight gain, alcohol use, accidents, and injuries [1–15]. Research also suggests that these adverse health effects vary in relation to the amount or duration of sleep problems [2–6, 12–15]. The US data show that, compared to children and adolescents who do not experience any sleep problems during the week, those who experience inadequate sleep during the entire week have 3-4 times higher risks of serious behavioral problems, 4-5 times higher risks of depression and anxiety, 2.5 times higher risk of ADD/ADHD, 3.2 times higher risk of migraine headaches, 1.5 times higher risk of being in fair/poor overall health, 1.6 times higher risk of repeating a grade or having a problem at school, and 2.8 times higher risk of missing >2 weeks of school during a year [16–18].

Past research has examined the impact of a number of sociodemographic and behavioral factors on childhood sleep problems [1, 2, 4, 19–23]. These factors include child's age, gender, race/ethnicity, household socioeconomic status (SES), and such behavioral risk factors as physical activity, television viewing, and recreational computer use [1, 2, 4, 19–23]. Although the effects of neighborhood factors have been examined for a number of child health and behavioral outcomes such as physical inactivity, obesity, school performance, perceived health status, mental health, behavioral problems, and youth violence [16, 24–31] few studies have addressed the relationship between neighborhood environments and children's sleep problems [1, 2, 32]. To our knowledge, the impact of neighborhood social conditions and built environments on sleep problems in the US has not been fully explored using a nationally representative sample of school-aged children.

Analyzing the health effects of neighborhood environment is important because neighborhood conditions reflect the broader social and community contexts within which variations in individual health and social behaviors occur [16, 24–27, 33]. Many aspects of neighborhood environment that are thought to influence child health and behavioral outcomes, such as socioeconomic deprivation, poor housing, crime, and lack of social amenities, are potentially modifiable through social policies [16, 24, 25, 34]. Additionally, neighborhood conditions have been linked to a variety of health and behavioral outcomes among both children and adults, including obesity and physical activity, infant mortality, low birthweight, smoking, self-rated health, mental health, injury, and mortality [16, 24–26, 33]. As such, improvements in neighborhood environment have the potential to positively impact a wide range of childhood health inequalities, including those in sleep problems [16, 26]. Emphasis on the neighborhood environment and broader social structure is also consistent with the *Healthy People 2020* objectives [35].

Besides neighborhood factors, examining the sleep effects of household SES, race/ethnicity, and behavioral characteristics is important as well because they identify additional opportunities to reduce health disparities among children through targeted interventions. Moreover, health-related behaviors, which are amenable to change through public policy and social interventions, are one possible mechanism through which neighborhood, ethnic, and social factors might influence sleep patterns in children.

The National Survey of Children's Health (NSCH) allows us to explore the association between neighborhood conditions, household SES, behavioral risk factors, and childhood sleep problems in the US. In this study, we (1) examine trends in prevalence of sleep problems by child's age, gender, and race/ethnicity, (2) estimate prevalence of sleep problems by a variety of neighborhood, household, and child-level characteristics, (3) assess whether neighborhoods effects on sleep problems persist after adjusting for household SES and sociodemographic characteristics, (4) examine the potential intervening mechanisms, particularly behavioral factors of physical activity, recreational screen time, and exposure to secondhand smoke (SHS), through which neighborhood conditions may influence sleep patterns, and (5) examine whether the sleep effects of neighborhood environment and behavioral factors vary by child's age and gender.

## 2. Methods

 Trends in prevalence of sleep problems by age, gender, and race/ethnicity were analyzed using the 2003, 2007, and 2011-2012 NSCH [18, 36–41]. However, data for the detailed analyses of neighborhood, socioeconomic, and behavioral determinants came from the 2007 NSCH because it had the most complete information on covariates, including composite neighborhood indices [18, 36, 37, 40]. All three rounds of the survey were conducted by the National Center for Health Statistics (NCHS), with funding and direction from the Maternal and Child Health Bureau [36–39]. The purpose of the NSCH was to provide national and state-specific prevalence estimates for a variety of children's health and well-being indicators [36–39]. The surveys included an extensive array of questions about children's health and the family, including parental health, stress and coping behaviors, family activities, and parental concerns about their children [18, 36–41]. Interviews were conducted with parents, and special emphasis was placed on factors related to children's well-being.

 All three rounds of the NSCH were cross-sectional telephone surveys. The 2003 survey was conducted between January 2003 and July 2004; the 2007 survey was conducted between April 2007 and July 2008; and the 2011-2012 survey was conducted between February 2011 and June 2012 [18, 36–41]. The sample size was 102,353 children <18 years of age for the 2003 survey, 91,642 for the 2007 survey, and 95,677 for the 2011-2012 survey. In each survey, the average sample size was about 1,800-2,000 children per state [18, 36–41]. In all three rounds of the survey, a random-digit-dial sample of households with children <18 years of age was selected from each of the 50 states and the District of Columbia. One child was selected from all children in each identified household to be the subject of the survey [18, 36–41]. Interviews were conducted in English, Spanish, and four Asian languages. The respondent was the parent or guardian who knew most about the child's health status and health care. All survey data were based on parental reports. The interview completion rate for the NSCH, measuring the percentage of completed interviews among known households with children, was 68.8% in 2003, 66.0% in 2007, and 54.1% for the landline sample and 41.2% for the cell-phone sample in 2011-2012 [37–39]. The overall response rate at the national level was 55.3% in 2003 and 46.7% in 2007 [37, 38].The overall response rate for the 2011-2012 survey is not yet available. Substantive and methodological details of the 2003, 2007, and 2011-2012 surveys are described elsewhere [36–41]. The NCHS Research Ethics Review Board approved all data collection procedures for each round of the survey.

 The sample size for the detailed covariate analysis, based on the 2007 NSCH, was 63,352 children and adolescents aged 6–17. The dependent variable, sleep problems, was based on the question “During the past week, on how many nights did the child get enough sleep for a child his/her age?” From this question, we derived two measures of inadequate sleep: children who experienced <7 days/week of adequate sleep (or at least 1 day/week of inadequate sleep) and those who experienced <5 days/week of adequate sleep (or at least 3 days/week of inadequate sleep) [16, 17, 42]. The latter measure, representing more serious sleep problems, tends to capture the amount of sleep problems and may be clinically more relevant as it leads to stronger physical and mental health effects [16, 17, 42]. 

Neighborhood social conditions and built environments were the primary covariates of interest. Neighborhood social conditions included dichotomous measures of perceived neighborhood safety, presence of garbage/litter in the neighborhood, poor/dilapidated housing, and vandalism such as broken windows or graffiti [26]. We used a previously developed factor-based index of neighborhood social conditions that combined the above four neighborhood social indicators with respective factor loadings or weights of 0.52, 0.69, 0.71, and 0.72 [26]. Higher scores on the neighborhood social conditions index (alpha = 0.57) represent more favorable socioeconomic conditions. The built environment index, developed previously, consisted of 4 variables: neighborhood access to sidewalks/walking paths; parks/playgrounds; recreation or community centers; and library/bookmobile, with respective factor loadings of 0.69, 0.75, 0.66, and 0.67 [26]. Higher scores on the built environment index (alpha = 0.64) represent higher levels of health-promoting neighborhood amenities. Both indices were standardized to have a mean score of 100 and standard deviation of 20. Note that the two neighborhood indices were orthogonal or independent of each other [26]. Two indicators of household SES were used: parental education and household income/poverty levels. 

**Figure 1:**
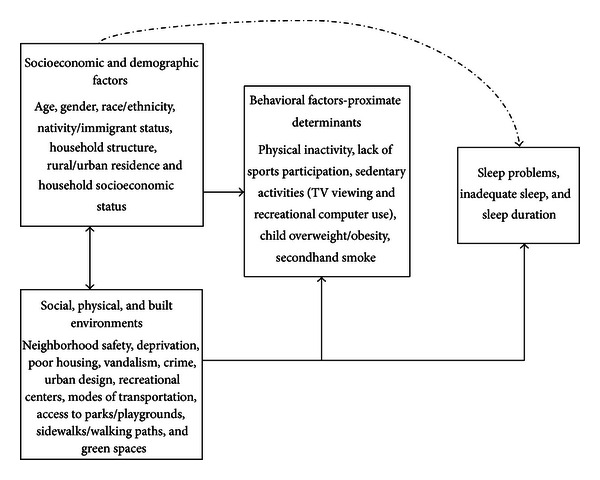
A simple model of neighborhood and sociobehavioral determinants of sleep problems in children and adolescents.

We used social determinants of health framework to model links between neighborhood conditions, household socioeconomic characteristics, health-related behaviors, and childhood sleep problems (Figure 1) [16, 25, 26, 28, 43, 44]. Within this framework, neighborhood and household socioeconomic characteristics are considered underlying determinants, [16, 25, 26, 28, 43, 44] which may influence sleep patterns by creating conditions (e.g., noise, violence, and anxiety) that lead to sleep disturbance in children. They are also hypothesized to affect sleep problems indirectly through their effects on intervening psychosocial and behavioral mechanisms such as familial stress and behavioral risk factors such as physical activity, television viewing, alcohol, tobacco and substance use, and SHS exposure [16, 26, 28]. A bidirectional relationship between household SES and neighborhood conditions is postulated as neighborhood social and built environment conditions can influence household or individual education and income attainment, employment status, and housing tenure. On the other hand, age and racial/ethnic composition, household socioeconomic conditions, and place of residence can contribute significantly to the makeup of the neighborhoods, community economic development, and the kinds of social and physical amenities that might be available to neighborhood or community residents [16, 26, 28, 43, 44]. 

Using this framework and past research as a guide, we considered twelve covariates of childhood sleep problems, in addition to the neighborhood conditions. These included child's age, gender, race/ethnicity, nativity/immigrant status, household composition, metropolitan/non-metropolitan residence, household/parental education (<12, 12, 13–15, ≥16 years), household poverty status measured as a ratio of family income to the poverty threshold (<100%, 100–199%, 200–399%, ≥400%), television viewing, recreational computer use, physical activity levels, and SHS exposure [1, 2, 4, 16, 19–23, 26, 28]. SHS exposure was determined by whether anyone smoked inside child's home. All other covariates were measured as shown in Tables 1–4.

Income was imputed for 9% of the observations by using a multiple imputation technique [37]. For all other covariates, there were few or no missing cases, which were excluded from the multivariate analyses.

 The *χ* ^2^ statistic was used to test the overall association between covariates and sleep problems. The *t*-statistic was used to test the difference in prevalence between any two groups or time points. Logistic regression was used to examine the association between neighborhood and behavioral characteristics and sleep problems, after adjusting for the above covariates. To account for the complex sample design of the NSCH, SUDAAN software was used to conduct all statistical analyses [45].

## 3. Results

### 3.1. Trends in the Prevalence of Sleep Problems, 2003–2012

 The prevalence of sleep problems increased significantly between 2003 and 2012 (Table 1). The proportion of children with <7 days/week of adequate sleep increased from 31.2% in 2003 to 35.7% in 2007 and 41.9% in 2011-2012, whereas the prevalence of adequate sleep <5 days/week rose from 12.6% in 2003 to 13.6% in 2011-2012. The number of children aged 6–17 with at least 1 day/week of sleep problems rose from an estimated 15.1 million in 2003 to 20.5 million in 2011-2012. The increase in prevalence of sleep problems was more pronounced among children aged 6–11 and females. During 2003–2012, while children in all racial/ethnic groups experienced a marked increase in sleep problems at least 1 day/week, the prevalence of serious (≥3 days/week) sleep problems increased significantly only among white, mixed-race, and “other” children (Table 1).

### 3.2. Neighborhood and Sociobehavioral Disparities in Sleep Problems, 2007

Descriptive characteristics of the 2007 sample are shown in Table 2. Approximately 9% of the child population lived in neighborhoods with the most-unfavorable social or built environments. Non-Hispanic white children were the largest racial/ethnic group (56.5%), followed by Hispanics (18.9%), blacks (14.8%), and Asians (3.3%). Approximately 17% of children lived below the poverty line, and 8.5% of children had parents who had less than a high school education. Approximately 22% of children watched television >2 hours/day, while 10.2% of children were physically inactive and 8.9% were exposed to secondhand smoke.  Table 3 shows observed prevalence of childhood sleep problems in 2007 according to various neighborhood, sociodemographic, and behavioral characteristics. The prevalence of sleep problems varied significantly in relation to neighborhood socioeconomic and built-environmental characteristics. Approximately 10% of children in neighborhoods with the most-favorable social environment had serious sleep problems, compared with 16.2% of children in neighborhoods with the least-favorable social environment. Children living in unfavorable neighborhoods that were characterized by safety concerns, garbage/litter in streets/sidewalks, poor/dilapidated housing, or vandalism had 19%–29% higher prevalence of serious sleep problems than those in more favorable neighborhoods (Table 3). 

Approximately 15.7% of children in neighborhoods with the fewest health-promoting amenities had serious sleep problems, compared with 12.6% of children in neighborhoods with the most health-promoting amenities (Table 3). Specifically, children living in neighborhoods with no access to parks and playgrounds had 8% and 18% higher risks of ≥1 day/week and ≥3 days/week of sleep problems, respectively, than those with access to these amenities.

The prevalence of sleep problems was positively associated with child's age. Approximately 47% of children aged 15–17 experienced at least 1 day/week of sleep problems, compared to 26.1% of children aged 6–8. The prevalence of more serious sleep problems was 3.4 times greater among older adolescents compared to younger children. Hispanic and Asian children had fewer sleep problems than white and black children, whereas children of immigrant parents had fewer sleep problems than those with US-born parents (Table 3). Higher parental education and income were associated with a higher prevalence of at least 1 day/week of sleep problems.

In terms of behavioral effects, higher levels of physical inactivity, recreational computer use, and SHS exposure were significantly associated with both ≥1 day/week and ≥3 days/week of sleep problems. Higher levels of television viewing were associated with only more serious sleep problems (Table 3).

Since the adjusted effects of neighborhood factors, household SES, and demographic factors were generally similar in both the sociodemographic and full sociobehavioral models, we only interpret the results from the full multivariate models in Table 4. Higher risks of sleep problems associated with unfavorable neighborhood social conditions persisted even after the adjustment of sociodemographic and behavioral characteristics. Children in neighborhoods with the most unfavorable social conditions had 35% and 43% higher adjusted odds of ≥1 day/week and ≥3 days/week of sleep problems, respectively, than their counterparts from the most-favorable neighborhood social environment. Each of the specific neighborhood social conditions was significantly related to both sleep measures. Children in neighborhoods with safety concerns, garbage/litter, poor/dilapidated housing, and vandalism had 40%, 29%, 26%, and 24% higher adjusted odds of serious sleep problems than children in neighborhoods without these unfavorable conditions, respectively (Table 4).

 The built environment index was only significantly related to more serious sleep problems. Children in neighborhoods with the fewest health-promoting amenities had 37% higher adjusted odds of serious sleep problems than children in neighborhoods with the most amenities. Not having an access to parks/playgrounds or a library/bookmobile was associated with 20%–24% higher adjusted odds of serious sleep problems. While there were no significant gender differentials in sleep problems, older adolescents aged 15–17 had, respectively, 2.4 and 3.4 times higher odds of ≥1 day/week and ≥3 day/week of sleep problems than children aged 6–8. No significant racial/ethnic differentials were found for serious sleep problems; however, non-Hispanic white and mixed-race children had 29% and 38% higher adjusted odds of experiencing at least 1 day/week of sleep problems than Hispanic children. Nativity remained a stronger risk factor, with children born to immigrant parents having 22%–29% lower odds of sleep problems than children of US-born parents. Children in single-mother households had 35% higher adjusted odds of serious sleep problems than those in two-parent households. Although household education or income was not significantly related to serious sleep problems, children from higher-SES households were significantly more likely to experience at least 1 day/week of sleep problems than children from lower-SES households (Table 4).

 Neighborhood environments and household SES partly accounted for the effects of behavioral factors on sleep problems. Children with no physical activity had 21% and 47% higher adjusted odds of ≥1 day/week and ≥3 day/week of sleep problems, respectively, than children who exercised at least 5 days/week. Children with >2 hours/day of recreational computer use had 20% and 41% higher adjusted odds of ≥1 day/week and ≥3 day/week of sleep problems, respectively, than children with <1 hour/day of computer use. Children exposed to SHS had 23% higher adjusted odds of serious sleep problems than those without exposure (Table 4). 

We also examined interaction models of neighborhood, household SES, and behavioral factors by child's age (6–11, 12–17) and gender. However, none of the interactions were statistically significant, and the effects of the covariates on sleep problems were similar for males and females and for younger and older children and adolescents (data not shown). 

## 4. Discussion

Sleep problems are increasingly being recognized as an important public health problem in the United States [3]. Our study showed a marked and consistent increase in the prevalence of childhood sleep problems between 2003 and 2012. Currently, half of all adolescents aged 15–17 and more than one-third of young children aged 6–8, and approximately 21 million school-aged children and adolescents in the US are reported to have at least 1 day/week of sleep problems. What might account for this substantial increase in prevalence? It is conceivable that changes in the social, built, or obesogenic environments, demographic composition of the population, and physical inactivity levels or other sedentary activities may have contributed to the rise in sleep problems among US children—but a more formal analysis of the 2003, 2007, and 2011-2012 National Surveys of Children's Health is needed to shed more light on the rising trend.

To our knowledge, our study is the first to examine variations in sleep problems according to a variety of neighborhood and behavioral factors using a large, nationally representative sample of school-aged children and adolescents. In addition to neighborhood influences, assessing effects of screen time, physical inactivity, and SHS exposure on sleep problems in children represented an important aspect of our study. Increased risks of sleep problems associated with excess television viewing, recreational screen time, physical inactivity, and SHS exposure were independent of neighborhood conditions and household SES, and are consistent with those reported previously in the US and international studies [1, 4, 19, 20, 22, 23]. 

Neighborhood effects reported here are consistent with limited research that shows higher risks of sleep problems in children associated with unsafe school or neighborhood environment and greater area-based neighborhood distress or socioeconomic disadvantage [1, 2, 32]. In our study, the association between neighborhood factors and sleep problems was not explained or mediated by household SES and behavioral characteristics. Thus, most of the neighborhood effects reported here appear to be either direct or operate through psychosocial or behavioral mechanisms (such as parental stress, family conflict, family cohesiveness, social support, alcohol and substance use) that we did not consider in our analysis. 

While neighborhood effects on childhood obesity, physical activity, and mental health have been shown to vary according to child's age and gender, [16, 24, 26, 28] we did not find similar patterns for sleep problems. Thus, when it comes to sleep problems, boys and girls as well as younger and older children appear to be equally vulnerable to unfavorable neighborhood environments. 

Higher prevalence of sleep problems in children from higher-SES households is consistent with the patterns observed previously for children and adults in the US and elsewhere [1, 2, 19, 46]. However, some studies have shown an inverse association between SES and sleep problems [4, 20, 47]. The inconsistent SES patterns in sleep behavior across studies may partly be due to differences in sleep measures and data sources [1]. 

A major strength of our study includes estimating the effects of a variety of neighborhood conditions and composite indices of neighborhood environment on children's sleep problems. Another important contribution of this study is the concurrent evaluation of the impact of both neighborhood factors and health behaviors on sleep problems. Although many features of the neighborhood environment may directly lead to sleep problems (such as noise, violence, and safety fears), we have identified possible casual pathways such as excessive television viewing, physical inactivity, recreational screen time, and SHS exposure which are potentially modifiable through public health policies. Examining specific features of the neighborhood environment brings us closer to intervention (e.g., better amenities, built environments, neighborhood revitalization, crime reduction, affordable housing, community safety, and safe streets) that could lead to better sleep health. The other strengths of our study include the large sample size, the generalizability of our findings, and examination of whether sleep effects of neighborhood conditions, household SES, and behavioral factors vary by age and gender. 

This study has limitations. Children's behavioral measures, including sleep behavior, in the NSCH were based on parental reports and may not accurately reflect the true prevalence, particularly among older adolescents. However, prevalence of inadequate sleep reported here is consistent with that reported in other epidemiologic studies [1, 48, 49]. Moreover, previous research has indicated self- or parental reports to be reliable and valid reports of children's sleep patterns and disturbances and has shown satisfactory agreement between objective measures such as actigraphy and parent-report or survey-based measures [50–54]. Second, although neighborhood characteristics considered in our study are important measures of the social environment, they are perceived or parent-reported measures. While subjective ratings of the neighborhood environment may result in underestimation of the neighborhood effects on sleep health, both subjective and objective measures of the neighborhood environment are needed [26, 33]. Third, same-source bias is a possible limitation since neighborhood conditions and sleep problems were reported by the same respondents [16, 55]. The effects of neighborhood conditions on sleep problems could have been underestimated if disadvantaged individuals provided a more positive assessment of neighborhood environment [16, 33, 55]. Individuals in disadvantaged neighborhoods may be more optimistic about their neighborhood situation and, consequently, may downgrade the severity of problems facing their neighborhood surroundings, a phenomenon called “psychological adjustment” [16, 33, 55]. Fourth, our sleep measures were based on parental response to a single question regarding adequacy of child's sleep. No information in the survey was available about sleep quality, sleep duration, and types of sleep problems such as obstructive sleep apnea, difficulty falling or staying asleep through the night, and daytime sleepiness. Fifth, because of the cross-sectional nature of the NSCH, causal inferences about the relationships between neighborhood environment, household SES, behavioral factors, and childhood sleep problems cannot be drawn [16, 17, 26]. Sixth, as with most sample surveys, the potential for nonresponse bias exists for the NSCH, implying that the sample interviewed differed from the targeted child population in a systematic fashion [37]. Since response rates in the NSCH tend to be lower in urban areas and low-income and ethnic-minority populations, differential nonresponse bias might affect (most likely underestimate) the impact of neighborhood disadvantage, individual SES, and race/ethnicity on sleep problems [37]. However, the nonresponse adjustment to the sampling weights in the NSCH might have reduced the potential magnitude of these biases [37]. Lastly, the increased use of cell/mobile phone use in recent years, especially among young, minority, renters, and low-income adults, may be an additional source of noncoverage bias for landline only surveys such as the 2007 NSCH [56, 57].

In conclusion, the evidence presented here suggests that favorable neighborhood conditions and positive health behaviors are significantly associated with reduced risk of sleep problems in children, which, in turn, may support reductions in overall child health inequalities given the wide-ranging health effects of poor sleep. While behavioral changes such as increased physical activity, reduced television viewing and computer use, and reduced exposure to secondhand smoke can be beneficial in promoting children's sleep health, social policy measures aimed at improving the broader social and physical environments can be vital to improving overall child health in general and their sleep health in particular. Continued surveillance and monitoring of the prevalence of childhood sleep problems as well as its determinants are essential in order to better understand the role of broader societal factors and health behaviors and to design effective public health interventions, including public awareness and educational campaigns [46].

## Figures and Tables

**Table 1:** Trends in weighted prevalence (%) of sleep problems among US children aged 6–17, 2003–2012: the National Survey of Children's Health.

													% change in prevalence	
	2011-2012		2007		2003		2011-2012		2007		2003		during 2003–2012	
	<5 days/week		<5 days/week		<5 days/week		<7 days/week		<7 days/week		<7 days/week		<5 d/w of	<7 d/w of
	of adequate sleep		of adequate sleep		of adequate sleep		of adequate sleep		of adequate sleep		of adequate sleep		adequate	adequate
	%	SE	%	SE	%	SE	%	SE	%	SE	%	SE	sleep	sleep
Total population	13.63	0.30	13.33	0.32	12.58	0.23	41.85	0.44	35.69	0.47	31.24	0.31	8.35^a^	33.96^a^
Child's age (years)														
6–8	7.82	0.48	6.68	0.43	7.49	0.42	34.43	0.85	26.06	0.89	22.81	0.59	4.41	50.94^a^
9–11	10.39	0.55	9.03	0.64	7.91	0.37	40.64	0.89	29.13	0.92	26.13	0.60	31.35^a^	55.53^a^
12–14	13.94	0.62	14.53	0.68	12.38	0.43	42.07	0.88	40.08	0.94	33.12	0.61	12.60^a^	27.02^a^
15–17	22.10	0.70	22.71	0.76	22.69	0.55	49.97	0.88	46.83	0.95	42.82	0.64	−2.60	16.70^a^
Child's sex														
Male	13.23	0.42	12.75	0.44	12.48	0.31	41.07	0.61	35.45	0.65	31.00	0.43	6.01	32.48^a^
Female	14.05	0.42	13.94	0.48	12.68	0.33	42.66	0.63	35.94	0.68	31.49	0.45	10.80^a^	35.47^a^
Race/ethnicity														
Hispanic	11.63	0.80	10.82	0.92	13.88	0.74	36.34	1.23	28.54	1.39	25.23	0.91	−16.21^a^	44.03^a^
Non-Hispanic white	14.11	0.35	13.80	0.39	12.27	0.24	45.18	0.50	39.40	0.55	33.87	0.35	15.00^a^	33.39^a^
Non-Hispanic black	14.80	0.83	15.31	0.85	13.58	0.71	40.82	1.20	32.75	1.11	29.10	0.91	8.98	40.27^a^
Non-Hispanic mixed race	15.63	1.34	12.70	1.27	13.20	1.44	43.26	1.82	38.66	2.46	31.03	1.78	18.41^a^	39.41^a^
Other^b^	12.78	1.21	12.36	1.39	9.39	1.07	35.69	1.72	28.71	1.91	24.91	1.60	36.10^a^	43.28^a^

^a^The *t*-test for change in prevalence between 2003 and 2012 was statistically significant at *P* < 0.05. ^b^includes Asians and Pacific Islanders and American Indians/Alaska Natives.

**Table 2:** Descriptive statistics of the sample for children aged 6–17 years, according to neighborhood, sociodemographic, and behavioral characteristics: the 2007 National Survey of Children's Health (*N* = 63,352).

Sociodemographic and behavioral characteristics	Unweighted number in sample	Weighted percent in sample
Index of neighborhood socioeconomic conditions		
20.78–67.09 (least favorable)	4,618	8.88
67.10–88.32	4,187	7.31
88.33–104.99	10,635	17.62
105.00–111.40 (most favorable)	42,981	66.19
Neighborhood safety		
Safe	56,356	86.57
Unsafe	6,328	13.43
Presence of garbage/litter in neighborhood		
Yes	9,427	16.04
No	53,348	83.96
Poorly kept or dilapidated/rundown housing in neighborhood		
Yes	8,717	14.21
No	53,981	85.79
Vandalism such as broken windows or graffiti in neighborhood		
Yes	6,007	11.17
No	56,757	88.83
Index of neighborhood built environment		
46.40–67.04 (low amenities)	5,121	8.53
67.05–81.39	8,227	12.02
81.40–104.99	20,306	32.40
105.00–116.40 (high amenities)	28,193	47.05
Neighborhood access to sidewalks or walking paths		
Yes	43,947	72.19
No	18,840	27.81
Neighborhood access to parks or playgrounds		
Yes	49,025	79.33
No	13,768	20.67
Neighborhood access to a recreation or community center		
Yes	39,915	64.95
No	22,132	35.05
Neighborhood access to a library or bookmobile		
Yes	54,594	86.33
No	8,122	13.67
Child's age (years)		
6–8	13,512	24.55
9–11	14,083	24.23
12–14	16,338	26.00
15–17	19,419	25.22
Child's sex		
Male	32,981	51.15
Female	30,371	48.85
Race/ethnicity		
Non-Hispanic white	43,444	56.53
Non-Hispanic black	6,363	14.81
Hispanic	7,245	18.85
American Indian/Alaska native	818	0.83
Asian	1,452	3.33
Hawaiian/Pacific Islander	309	0.34
Non-Hispanic mixed race	2,753	3.74
Other	968	1.56
Child's nativity/immigrant status		
Born to immigrant parents	7,964	19.02
Born to US-born parents	55,388	80.98
Household composition		
Two-parent biological	41,673	62.49
Two-parent stepfamily	5,984	10.12
Single mother	10,744	19.76
Other family type	4,951	7.63
Place of residence		
Metropolitan	43,859	83.69
Non-metropolitan	19,493	16.31
Household poverty status (ratio of family income to poverty threshold)		
Below 100%	6,886	17.08
100%–199%	10,500	20.51
200%–399%	21,624	32.17
At or above 400%	24,343	30.24
Highest household or parental education level (years)		
<12	3,666	8.47
12	10,263	23.53
13–15	17,813	26.87
16+	30,193	41.13
Television watching (number of hours per day)		
<1	13,282	20.66
1	19,176	29.07
2	18,252	28.41
>2	12,151	21.87
Recreational computer use (number of hours per day)		
<1	30,663	51.30
1-2	24,036	38.23
>2	6,352	10.47
Physical activity (number of days per week)		
0	5,649	10.21
1-2	7,571	12.36
3-4	15,529	23.95
5+	34,096	53.48
Secondhand smoke exposure		
Yes	5,293	8.93
No	57,620	91.07

**Table 3:** Weighted prevalence of sleep problems among US children aged 6–17 by neighborhood, sociodemographic, and behavioral characteristics: the 2007 National Survey of Children's Health (*N* = 63,352).

Sociobehavioral characteristic	Less than 5 days/week of adequate sleep			Less than 7 days/week of adequate sleep		
	%	SE	*P* value	%	SE	*P* value
Index of neighborhood socioeconomic conditions			<0.001			0.282
20.78–67.09 (least favorable)	16.20	1.16		38.20	1.70	
67.10–88.32	15.46	1.37		37.80	1.96	
88.33–104.99	15.77	0.85		35.44	1.08	
105.00–111.40 (most favorable)	12.10	0.38		35.32	0.57	
Neighborhood safety			<0.001			0.588
Safe	12.87	0.34		35.89	0.50	
Unsafe	16.62	1.01		35.10	1.36	
Presence of garbage/litter in neighborhood			<0.001			<0.001
Yes	16.41	0.88		39.33	1.17	
No	12.77	0.35		35.07	0.51	
Poorly kept or dilapidated/rundown housing in neighborhood			0.005			0.083
Yes	15.68	0.91		37.76	1.27	
No	12.95	0.35		35.41	0.51	
Vandalism such as broken windows or graffiti in neighborhood			0.030			0.192
Yes	15.58	1.10		37.68	1.58	
No	13.08	0.34		35.52	0.49	
Index of neighborhood built environment			0.049			0.575
46.40–67.04 (low amenities)	15.68	1.32		35.66	1.78	
67.05–81.39	14.68	0.88		37.32	1.21	
81.40–104.99	13.53	0.56		36.03	0.81	
105.00–116.40 (high amenities)	12.61	0.48		35.38	0.7	
Neighborhood access to sidewalks or walking paths			0.500			0.985
Yes	13.22	0.40		35.76	0.58	
No	13.68	0.56		35.78	0.79	
Neighborhood access to parks or playgrounds			0.009			0.025
Yes	12.90	0.35		35.18	0.52	
No	15.22	0.81		37.85	1.06	
Neighborhood access to a recreation or community center			0.120			0.557
Yes	13.02	0.40		36.03	0.58	
No	14.10	0.57		35.45	0.80	
Neighborhood access to a library or bookmobile			0.031			0.743
Yes	13.06	0.34		35.90	0.50	
No	15.39	1.03		35.41	1.39	
Child's age (years)			<0.001			<0.001
6–8	6.68	0.43		26.06	0.89	
9–11	9.03	0.64		29.13	0.92	
12–14	14.53	0.68		40.08	0.94	
15–17	22.71	0.76		46.83	0.95	
Child's sex			0.067			0.595
Male	12.75	0.44		35.45	0.65	
Female	13.94	0.48		35.94	0.68	
Race/ethnicity			0.040			<0.001
Non-Hispanic white	13.80	0.39		39.40	0.55	
Non-Hispanic black	15.31	0.85		32.75	1.11	
Hispanic	10.82	0.92		28.54	1.39	
American Indian/Alaska native	13.12	2.55		32.09	3.41	
Asian	12.04	2.13		26.96	2.94	
Hawaiian/Pacific Islander	13.90	3.92		36.17	7.86	
Non-Hispanic mixed race	12.70	1.27		38.66	2.46	
Other	12.32	2.40		29.01	3.02	
Child's nativity/immigrant status			<0.001			<0.001
Born to immigrant parents	10.33	0.79		27.08	1.2	
Born to US-born parents	14.04	0.35		37.71	0.49	
Household composition			<0.001			0.585
Two-parent biological	12.15	0.37		35.38	0.58	
Two-parent stepfamily	13.77	0.98		35.55	1.57	
Single mother	17.33	0.89		36.99	1.05	
Other family type	12.12	1.25		35.03	1.84	
Place of residence			0.102			0.064
Metropolitan	13.50	0.37		35.39	0.53	
Non-metropolitan	12.46	0.52		37.24	0.84	
Household poverty status (ratio of family income to poverty threshold)			0.494			<0.001
Below 100%	13.43	0.85		30.32	1.14	
100%–199%	12.94	0.76		32.29	1.06	
200%–399%	12.88	0.59		37.09	0.86	
At or above 400%	14.03	0.55		39.55	0.81	
Highest household or parental education level (years)			0.964			<0.001
<12	13.09	1.26		30.57	1.82	
12	13.73	0.77		34.29	1.07	
13–15	13.40	0.60		34.94	0.88	
16+	13.31	0.47		38.52	0.69	
Television watching (number of hours per day)			<0.001			0.996
<1	12.45	0.65		36.02	1.04	
1	12.64	0.61		35.82	0.87	
2	12.62	0.63		35.76	0.87	
>2	16.12	0.73		35.67	1.01	
Recreational computer use (number of hours per day)			<0.001			<0.001
<1	9.93	0.38		32.33	0.63	
1-2	15.89	0.60		39.03	0.79	
>2	20.93	1.17		43.05	1.59	
Physical activity (number of days per week)			<0.001			<0.001
0	18.77	1.14		37.83	1.55	
1-2	18.13	1.17		40.65	1.42	
3-4	12.76	0.57		36.24	0.90	
5+	11.27	0.41		33.79	0.64	
Secondhand smoke exposure			<0.001			0.042
Yes	17.88	1.07		38.76	1.43	
No	12.90	0.34		35.43	0.50	

*P* values associated with chi-square tests for independence between each covariate and sleep problems.

Both neighborhood indices have a mean score of 100 and a standard deviation of 20.

**Table 4:** Logistic regression models showing covariate-adjusted odds of sleep problems among US children aged 6–17 by neighborhood, sociodemographic, and behavioral characteristics: the 2007 National Survey of Children's Health.

Neighborhood conditions and Socio-behavioral characteristics	Less than 5 days/week of adequate sleep						Less than 7 days/week of adequate sleep					
	Sociodemographic model			Full socio-behavioral model			Sociodemographic model			Full socio-behavioral model		
	Adj OR	95% CI		Adj OR	95% CI		Adj OR	95% CI		Adj OR	95% CI	
Index of neighborhood socioeconomic conditions												
20.78–67.09 (least favorable)	1.48	1.21	1.80	1.43	1.18	1.75	1.37	1.16	1.60	1.35	1.15	1.59
67.10–88.32	1.38	1.10	1.73	1.38	1.11	1.72	1.22	1.03	1.45	1.22	1.03	1.45
88.33–104.99	1.45	1.25	1.69	1.42	1.22	1.65	1.12	1.00	1.26	1.11	1.00	1.25
105.00–111.40 (most favorable)	1.00	Reference		1.00	Reference		1.00	Reference		1.00	Reference	
Neighborhood safety												
Safe	1.00	Reference		1.00	Reference		1.00	Reference		1.00	Reference	
Unsafe	1.44	1.21	1.72	1.40	1.17	1.66	1.21	1.05	1.39	1.19	1.04	1.37
Presence of garbage/litter in neighborhood												
Yes	1.33	1.14	1.54	1.29	1.12	1.50	1.32	1.18	1.47	1.30	1.16	1.46
No	1.00	Reference		1.00	Reference		1.00	Reference		1.00	Reference	
Poorly kept or dilapidated/rundown housing in neighborhood												
Yes	1.28	1.09	1.50	1.26	1.09	1.48	1.20	1.07	1.35	1.19	1.06	1.34
No	1.00	Reference		1.00	Reference		1.00	Reference		1.00	Reference	
Vandalism such as broken windows or graffiti in neighborhood												
Yes	1.24	1.03	1.50	1.24	1.03	1.49	1.20	1.04	1.39	1.20	1.04	1.39
No	1.00	Reference		1.00	Reference		1.00	Reference		1.00	Reference	
Index of neighborhood built environment												
46.40–67.04 (low amenities)	1.39	1.11	1.74	1.37	1.10	1.70	1.06	0.89	1.26	1.05	0.89	1.25
67.05–81.39	1.20	1.01	1.43	1.21	1.02	1.44	1.05	0.92	1.19	1.05	0.93	1.19
81.40–104.99	1.14	1.00	1.30	1.14	1.00	1.29	1.05	0.96	1.15	1.05	0.96	1.15
105.00–116.40 (high amenities)	1.00	Reference		1.00	Reference		1.00	Reference		1.00	Reference	
Neighborhood access to sidewalks or walking paths												
Yes	1.00	Reference		1.00	Reference		1.00	Reference		1.00	Reference	
No	1.10	0.97	1.24	1.10	0.97	1.24	0.98	0.90	1.07	0.98	0.90	1.07
Neighborhood access to parks or playgrounds												
Yes	1.00	Reference		1.00	Reference		1.00	Reference		1.00	Reference	
No	1.21	1.05	1.40	1.20	1.04	1.38	1.07	0.96	1.18	1.06	0.96	1.18
Neighborhood access to a recreation or community center												
Yes	1.00	Reference		1.00	Reference		1.00	Reference		1.00	Reference	
No	1.13	1.01	1.27	1.11	0.99	1.25	1.00	0.92	1.09	1.00	0.91	1.09
Neighborhood access to a library or bookmobile												
Yes	1.00	Reference		1.00	Reference		1.00	Reference		1.00	Reference	
No	1.24	1.05	1.47	1.24	1.05	1.47	1.05	0.92	1.20	1.05	0.92	1.20
Child's age (years)												
6–8	1.00	Reference		1.00	Reference		1.00	Reference		1.00	Reference	
9–11	1.38	1.12	1.71	1.33	1.08	1.64	1.20	1.05	1.36	1.18	1.04	1.34
12–14	2.40	2.02	2.85	2.13	1.79	2.54	1.95	1.73	2.20	1.86	1.64	2.10
15–17	4.06	3.45	4.78	3.37	2.83	4.01	2.54	2.25	2.86	2.35	2.06	2.67
Child's sex												
Male	1.00	Reference		1.00	Reference		1.00	Reference		1.00	Reference	
Female	1.11	0.99	1.24	1.08	0.96	1.21	1.02	0.94	1.11	1.00	0.92	1.09
Race/ethnicity												
Non-Hispanic white	1.17	0.92	1.49	1.18	0.92	1.51	1.28	1.08	1.51	1.29	1.09	1.53
Non-Hispanic black	1.16	0.90	1.50	1.11	0.86	1.45	0.98	0.81	1.18	0.98	0.81	1.18
Hispanic	1.00	Reference		1.00	Reference		1.00	Reference		1.00	Reference	
American Indian/Alaska native	1.06	0.65	1.75	1.08	0.67	1.76	0.97	0.68	1.38	0.98	0.69	1.40
Asian	1.21	0.78	1.87	1.17	0.76	1.82	0.94	0.67	1.31	0.92	0.65	1.29
Hawaiian/Pacific Islander	1.16	0.58	2.31	1.21	0.62	2.38	1.20	0.59	2.46	1.22	0.60	2.51
Non-Hispanic mixed race	1.06	0.77	1.46	1.07	0.78	1.48	1.35	1.04	1.74	1.38	1.06	1.78
Other	0.63	0.34	1.20	0.61	0.32	1.19	0.59	0.36	0.96	0.59	0.36	0.97
Child's nativity/immigrant status												
Born to immigrant parents	0.80	0.65	0.98	0.78	0.63	0.96	0.72	0.62	0.84	0.71	0.61	0.83
Born to US-born parents	1.00	Reference		1.00	Reference		1.00	Reference		1.00	Reference	
Household composition												
Two-parent biological	1.00	Reference		1.00	Reference		1.00	Reference		1.00	Reference	
Two-parent stepfamily	1.01	0.83	1.22	0.98	0.81	1.19	0.96	0.82	1.12	0.95	0.81	1.11
Single mother	1.39	1.16	1.65	1.35	1.13	1.60	1.12	1.00	1.27	1.11	0.99	1.26
Other family type	0.92	0.69	1.23	0.89	0.66	1.20	1.01	0.84	1.22	1.01	0.83	1.22
Place of residence												
Metropolitan	1.15	1.01	1.29	1.13	1.00	1.28	0.98	0.89	1.07	0.97	0.88	1.06
Non-metropolitan	1.00	Reference		1.00	Reference		1.00	Reference		1.00	Reference	
Household poverty status (ratio of family income to poverty threshold)												
Below 100%	0.93	0.74	1.16	0.88	0.70	1.10	0.78	0.66	0.93	0.77	0.65	0.91
100%–199%	0.86	0.71	1.04	0.83	0.69	1.00	0.79	0.69	0.91	0.79	0.69	0.90
200%–399%	0.89	0.77	1.03	0.87	0.75	1.01	0.93	0.84	1.03	0.92	0.83	1.02
At or above 400%	1.00	Reference		1.00	Reference		1.00	Reference		1.00	Reference	
Highest household or parental education level (years)												
<12	0.95	0.72	1.25	0.86	0.65	1.13	0.90	0.72	1.12	0.87	0.70	1.08
12	0.91	0.77	1.08	0.85	0.71	1.01	0.87	0.77	0.98	0.86	0.76	0.97
13–15	0.93	0.80	1.08	0.89	0.77	1.03	0.86	0.78	0.96	0.86	0.77	0.96
16+	1.00	Reference		1.00	Reference		1.00	Reference		1.00	Reference	
Television watching (number of hours per day)												
<1				1.00	Reference					1.00	Reference	
1				0.97	0.82	1.14				0.95	0.84	1.08
2				0.94	0.79	1.11				0.95	0.85	1.08
>2				1.07	0.89	1.28				0.91	0.79	1.05
Recreational computer use (number of hours per day)												
<1				1.00	Reference					1.00	Reference	
1-2				1.22	1.07	1.40				1.08	0.98	1.18
>2				1.41	1.17	1.69				1.20	1.02	1.41
Physical activity (number of days per week)												
0				1.47	1.22	1.77				1.21	1.04	1.41
1-2				1.45	1.21	1.75				1.30	1.13	1.49
3-4				1.07	0.93	1.22				1.08	0.98	1.19
5+				1.00	Reference					1.00	Reference	
Secondhand smoke exposure												
Yes				1.23	1.04	1.46				1.07	0.94	1.23
No				1.00	Reference					1.00	Reference	

Sociodemographic logistic models included child's age, sex, race/ethnicity, nativity, household composition, metropolitan/nonmetropolitan residence, household poverty and education levels, and neighborhood social conditions or built environments. The full logistic model included neighborhood conditions and sociodemographic and behavioral covariates.
